# 3D Bioprinted GelMA Based Models for the Study of Trophoblast Cell Invasion

**DOI:** 10.1038/s41598-019-55052-7

**Published:** 2019-12-11

**Authors:** Houzhu Ding, Nicholas P. Illsley, Robert C. Chang

**Affiliations:** 1 Stevens Institute of Technology, Department of Mechanical Engineering, Hoboken, NJ 07030 USA; 2 Hackensack University Medical Center, Department of Obstetrics and Gynecology, Hackensack, NJ 07601 USA

**Keywords:** Tissue engineering, Experimental models of disease

## Abstract

Bioprinting is an emerging and promising technique for fabricating 3D cell-laden constructs for various biomedical applications. In this paper, we employed 3D bioprinted GelMA-based models to investigate the trophoblast cell invasion phenomenon, enabling studies of key placental functions. Initially, a set of optimized material and process parameters including GelMA concentration, UV crosslinking time and printing configuration were identified by systematic, parametric study. Following this, a multiple-ring model (2D multi-ring model) was tested with the HTR-8/SVneo trophoblast cell line to measure cell movement under the influence of EGF (chemoattractant) gradients. In the multi-ring model, the cell front used as a cell invasion indicator moves at a rate of 85 ± 33 µm/day with an EGF gradient of 16 µM. However, the rate was dramatically reduced to 13 ± 5 µm/day, when the multi-ring model was covered with a GelMA layer to constrain cells within the 3D environment (3D multi-ring model). Due to the geometric and the functional limitations of multi-ring model, a multi-strip model (2D multi-strip model) was developed to investigate cell movement in the presence and absence of the EGF chemoattractant. The results show that in the absence of an overlying cell-free layer of GelMA, movement of the cell front shows no significant differences between control and EGF-stimulated rates, due to the combination of migration and proliferation at high cell density (6 × 10^6^ cells/ml) near the GelMA surface. When the model was covered by a layer of GelMA (3D multi-strip model) and migration was excluded, EGF-stimulated cells showed an invasion rate of 21 ± 3 µm/day compared to the rate for unstimulated cells, of 5 ± 4 µm/day. The novel features described in this report advance the use of the 3D bioprinted placental model as a practical tool for not only measurement of trophoblast invasion but also the interaction of invading cells with other tissue elements.

## Introduction

Three-dimensional (3D) bioprinting is an additive manufacturing technique that enables the deposition of biomaterial-encapsulated living cells in the fabrication of complex 3D structures. These are utilized as analog tissue constructs targeting *in vitro* cell-based models and therapeutic applications^1,2^. Typically, a prescribed toolpath pattern, in tandem with either a three-axis robotic arm or translational stage, is used to control the relative motion of an extruding print head through space and time. Among the well-established bioprinting techniques, microextrusion-based bioprinting (micro-EBB) is a prevailing method that has advantages including facile implementation, cost-effectiveness, cell distribution control, and moderate ambient conditions during materials processing^3–6^. In addition to the manufacturing process design, judicious selection of the bioink material is essential to the printability, shape fidelity, mechanical stiffness, and cell proliferative capacity^7–10^. Therefore, systematic investigation of bioink properties during printing is critical for any bioprinted model^11–13^. On this methodological basis, the rational design and fabrication of bioprinted models conferring targeted cellular or tissue functions can extend the analytical reach and relevancy of fundamental cell biological models.

Herein, we have used micro-EBB in the bioprinted placenta model for the purpose of formulating studies of trophoblast invasion into the uterus during pregnancy. This process involves the differentiation of anchored, placental epithelial cytotrophoblast cells into motile, extravillous trophoblast cells (EVT), followed by invasion through the uterine decidual layer and into the myometrium. The EVT invasion enables multiple processes supporting the developing pregnancy. These include maintenance of immunologic neutrality at the maternal-fetal interface and remodeling of the maternal spiral arteries to promote nutrient delivery to the fetus^14,15^.

One of the most challenging aspects of research into placental function is the analysis of cell-cell interaction at the maternal-fetal interface. Accepting that, other than localization studies, *in vivo* observational data from human pregnancies is of limited value, it is also true that most animal pregnancies are inadequate models of the human uteroplacental system. Primate models, while close in structure and function, can only be monitored for input and output or imaged during pregnancy, rendering the uteroplacental unit a “black box” from which function can only be inferred. Moreover, specific manipulation of cell populations at the interface is difficult to accomplish in an animal model and risks damage to mother and fetus.

A variety of *in vitro* models have been proposed to study cell-cell interaction at the interface. For example, placental or decidual tissue fragments have been used to investigate *in vivo* structures and cell-cell interactions. However, tissue degradation leads to a limited lifetime for these model**s**^16–19^. In addition, manipulation of individual cellular components within the tissue is extremely difficult. Multicellular co-culture is another commonly used model in which cells can be directly co-cultured or embedded in an extracellular matrix. However, this model is usually limited to two cell types and does not simulate the 3D environment. There have also been models combining cells and tissues^20,21^ however, in addition to the problem of tissue degradation, these are frequently designed around a very specific question, limiting their broader utility.

Models of more complex cellular structures such as organ spheroids or bioreactor-cultured cells have also been developed. Some are focused on the 3D environment and its role in cellular differentiation and organization^22–26^ and often concentrated on one cell type (usually trophoblast). Others are designed to examine cell-cell interactions^27–29^, usually concerned with the interaction of two cell types. These often recapitulate trophoblast-endometrial implantation events but fail to capture the structural complexities of the *in vivo* microenvironment. The trophoblast organoid model recapitulates cytotrophoblast (CTB) differentiation into syncytiotrophoblast (STB) and EVT^30,31^ however, the structure is inverted compared to the normal villous tree, with the STB in the center of the organoid. Investigation of EVT interaction with other cells in this model, especially primary cell preparations, is less feasible given the need to grow out the EVT from the organoid while at the same time maintaining culture of the interacting cells. The placenta-on-a-chip model^32,33^ is intended to simulate the interaction between maternal and fetal circulations. Like the organoids, a limitation of the placenta-on-a-chip model is the length of time required to establish cell-lined microfluidic channels while maintaining a stable, primary (e.g. decidual) culture in the matrix separating the channels. Moreover, the limited depth of the matrix and the continuing flows through the channels may wash out crucial agents involved in the cell-cell interactions between invading EVT and decidual cells. While offering key functionalities, both of these models suffer from restrictions in the context of stable cell-cell interaction platforms. To this end, we have applied our advanced EBB technique in modeling the placental for the study of extravillous trophoblast (EVT) invasion with the long-term goal of exploring trophoblast-decidual cell interactions in a 3D environment. Based on our prior experience with trophoblast invasion in a 3D bioprinted multi-ring model, we undertook research to develop this model for practical studies of trophoblast invasion, using the HTR8/SVneo cell line, a model for invasive extravillous trophoblast. In this paper, specifically, we performed integrated parametric studies that not only identified optimal material and processing parameters for GelMA bioprinting but also included assessment of biological parameters such as cell spreading. These studies were a prelude to testing the design of two constructs, a multi-ring model^34^ and a multi-strip model. These were tested with or without the presence of an overlying GelMA layer to simulate 2D and 3D microenvironments (respectively) for cell invasion.

## Results

Based on the previous cell invasion study^35^, we outlined a study to measure trophoblast cell invasion in a bioprinted model using HTR8/SVneo, the extravillous trophoblast model cell line. We adopted GelMA as the base construct material and the multi-ring model as the starting point. However, during initial invasion experiments we became aware that cells within the hydrogel were relatively static, while cells at or close to the surface of the hydrogel showed a significant degree of invasion. Measurement of invasion rates for cells close to the hydrogel surface was deemed inappropriate as it did not appear to reflect the 3D environment. Accordingly, we proceeded to explore the optimization of cell invasion by the HTR8/SVneo cells, examining three key parameters, GelMA polymer concentration, hydrogel elastic modulus and cell viability. The first part of the results section is concerned with the parametric analysis of these contributing parameters. These elements were examined individually and then integrated to generate an optimization metric which takes into account these variables, yielding the most appropriate bioprinting parameters.

Specifically, the criteria for material selection are the combination of cell spreading degree (*D*_*spread*_), normalized cell viability (V) and GelMA shape fidelity (S, defined as normalized elastic compliance, reciprocal of compressive modulus) that maximize the capability of cell movement in 3D environment. Thus, the optimization problem is defined as:

1 $$\mathop{{\max }}\limits_{c,t}({w}_{1}S(c,t)+{w}_{2}V(c,t)+{w}_{3}{D}_{spread}(c,t))$$

$$Subject\,to\,c\in [3 \% ,5 \% ,10 \% ],t\in [15s,30s,45s],$$

where the *w*_1_, *w*_2_, *w*_3_ are the weights for the 3 terms in equation (1), with

$${w}_{1}+{w}_{2}+{w}_{3}=1\,and\,{w}_{i} > 0,for\,i=1,2,3$$

Based on different applications, *w*_*i*_ can be set or tested at different levels. As the intention was to investigate cell invasion in the 3D GelMA model, the requirement for GelMA model compliance (*S*), cell viability (*V*) and spreading level (*D*_*spread*_) are assumed equally important, thus *w*_1_ = *w*_2_ = *w*_3_ = 1/3. The definition and measurement of S, V and *D*_*spread*_ are described in the following sections.

### Parametric study of bioprinted GelMA model

**Figure 1:**
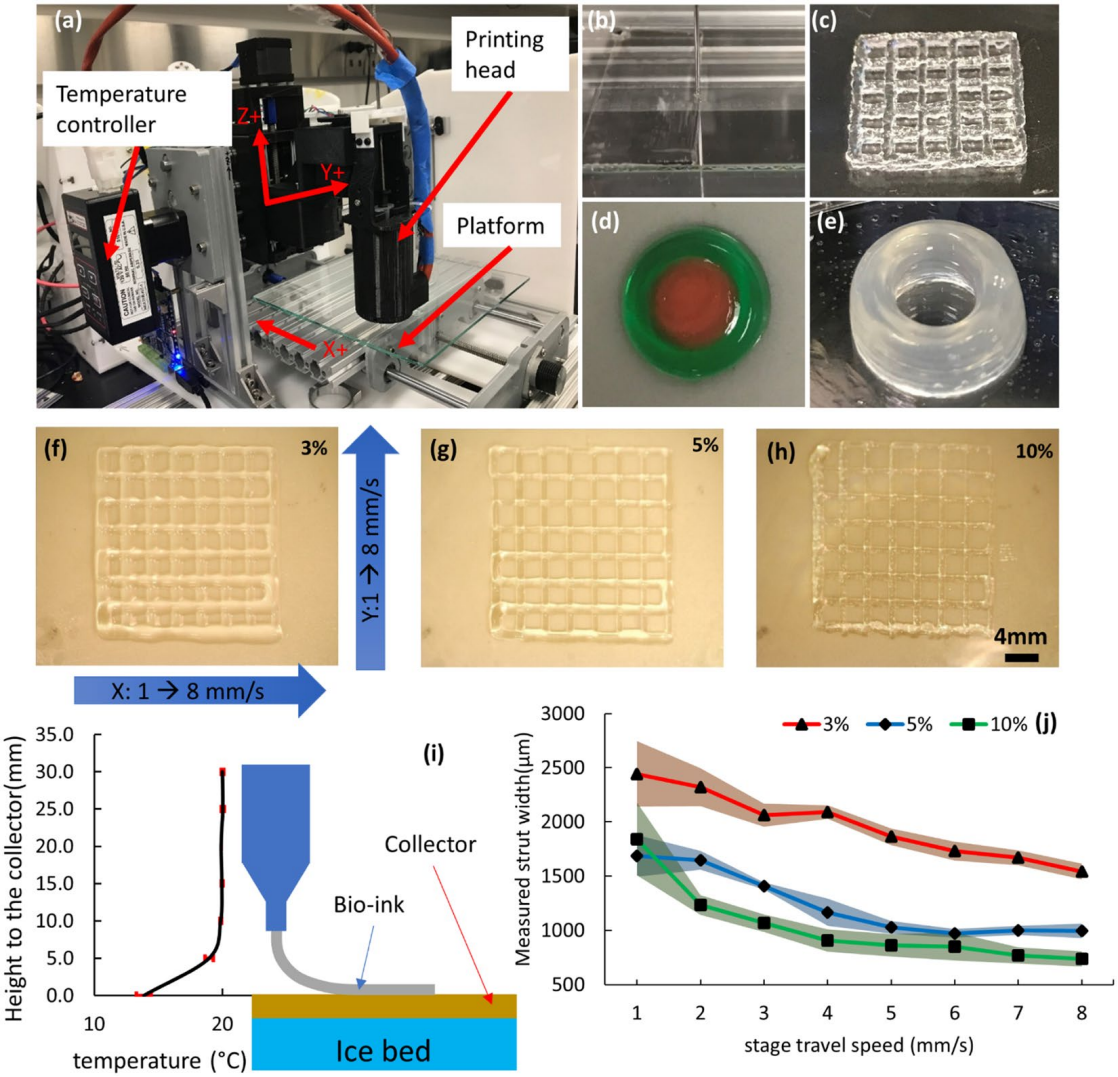
(**a**) Bioprinting system configuration. (**b–e**): Various bioprinted 3D constructs including single layer sheet, lattice structure, double ring structure and tubular structure. (**f–h**) Grid structures printed at different GelMA concentration with stage travel speed ranges from 1–8 mm/s at both x and y direction. (**i**) Schematic shows an ice bed put underneath the collector to allow thermal gelation of GelMA bioink before UV crosslinking, temperature gradient is measured and plotted. (**j**) Measured mean strut width with uncertainty of samples from (**f–h**).

A systematic study of several key bioprinting parameters, including GelMA polymer concentration, UV crosslinking time, and stage travel speed contributing to optimal GelMA model-printing parameters sets is described here. Figure 1(a) shows the customized bioprinting system configuration. Figure 1(b–e) show several bioprinted 3D constructs including single layer sheet, lattice structure, double ring structure and tubular structure, demonstrating the functionality of our customized extrusion bioprinter. Figure 1(f–h) are three grid structures printed at different GelMA concentrations with stage travel speeds varying from 1–8 mm/s along the direction of both the x- and y-axis. Due to the low viscosity, 3% (w/v) GelMA exhibits low printability at room temperature (around 22 °C). In order to print GelMA at low concentrations, an ice bed (schematically shown in Fig. 1(i)) is placed underneath the collector (glass cover-slide) to allow thermal gelation of GelMA bio-ink during printing (before UV crosslinking). The collector can be maintained at around 15 °C (measured by thermocouple) for about 20 min, which allows printing of multiple samples for the study. Not only 3% w/v GelMA but also 5%, and 10% GelMA can be printed onto the collector with the ice bed and maintain their shape during printing. From the perspective of printability, the Fig. 1(j) is the measured mean strut width of samples from (f)-(h) with an error band formed by standard deviations. These experiments define the printing conditions for GelMA at concentrations of 3–10%.

**Figure 2:**
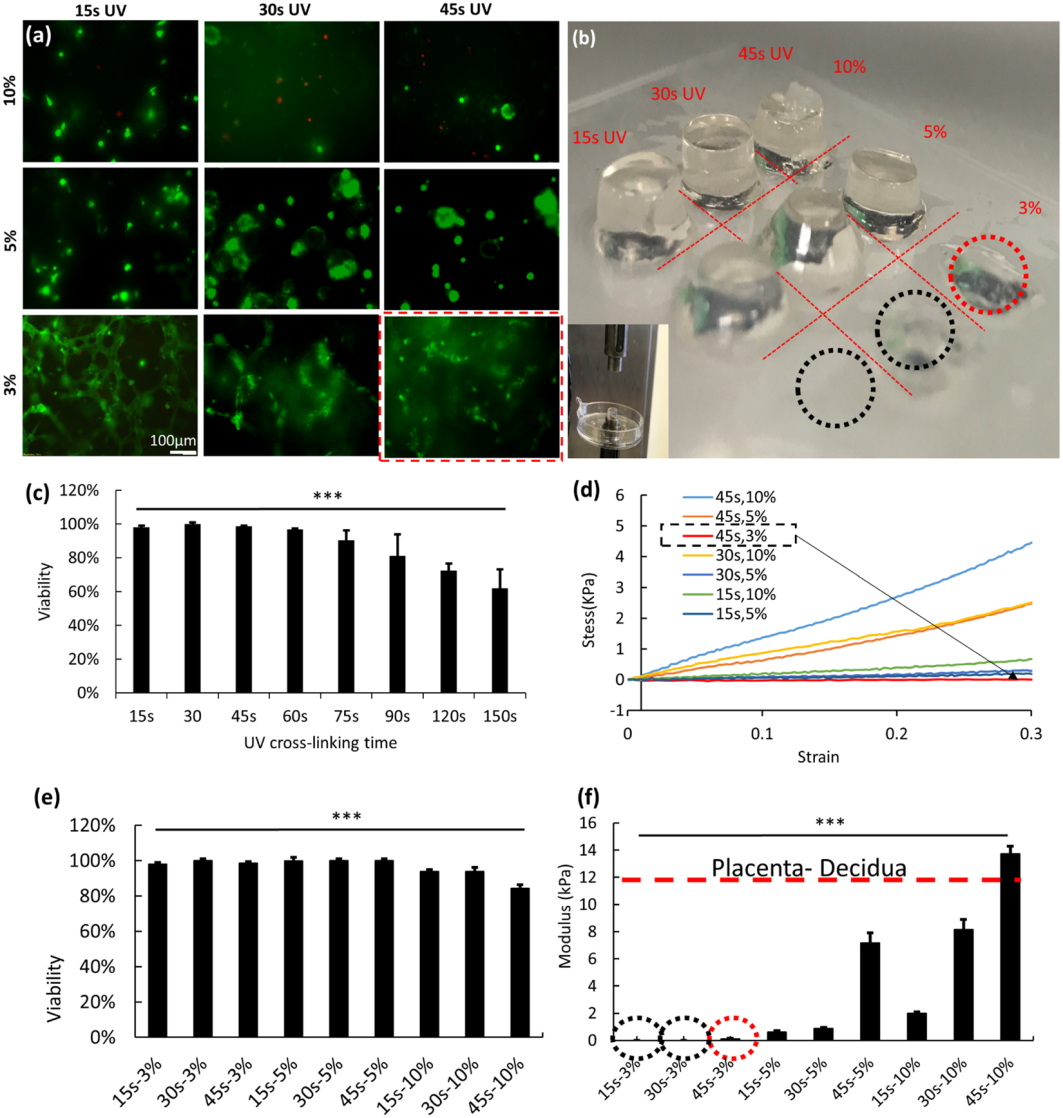
(**a**) Fluorescent images of live-dead cells in GelMA samples printed and crosslinked under conditions of differing GelMA concentration and UV crosslinkisng time. (**b**) GelMA pillars printed at 3%, 5%, 10% concentration and crosslinked at 15, 30 and 45 s UV curing time. (**c**) The viability of HTR-8/SVneo cells after printing as a function of UV crosslinking time. (**d**) The GelMA stress-strain curve measured from the samples described in (**b**). (**e**) Viability measurement of the 9 samples in (**a**). (**f**) Compressive modulus of samples in (**b**) with a value for the placenta-decidua taken from^34^.

The next step was to evaluate the shape fidelity, cell viability and cell morphology within the hydrogel. The shape fidelity is determined by cell-laden GelMA model stiffness (compressive modulus, or compliance, the reciprocal of elastic modulus). In Fig. 2, the optimal printing condition is evaluated by comparing cell viability, cell morphology and GelMA stiffness at various conditions.

Figure 2(a) is the fluorescent images of live-dead cells in GelMA samples printed and crosslinked at concentrations of 3–10% (w/v) and using a UV curing time of 15–45 s. Figure 2(b) illustrates GelMA pillars that are printed (into a 96 well plate) at different concentrations (3%, 5%, 10% (w/v)) and crosslinked under different UV curing times (15, 30, 45 s). The dimensions of the pillars in Fig. 2(b) are much larger than the actual construct size (height = 1 mm) for the invasion models. However, the larger pillar structures are amenable to compression testing (Fig. 2(d)) and serve to illustrate the nature of the construct material at different GelMA concentrations and crosslinking times. Figure 2(c) shows the viability of HTR-8/SVneo cells after printing as a function of UV cross-linking time; as crosslinking time increases, cell viability decreases. Figure 2(d) is the stress-strain curve of the 7 samples shown in Fig. 2(b). The two missing samples (3% (w/v) @ 15 s, 3% (w/v) @ 30 s) had shape fidelity too poor to maintain structure and permit compression testing (black dashed circle). Figure 2(e) plots the cell viability as a function of both GelMA concentration and UV crosslinking time, corresponding to the images in Fig. 2(a). Figure 2(f) plots the compressive modulus of samples in Fig. 2(b), where the red dashed circle is the optimal GelMA concentration with minimal measurable compressive modulus (largest compliance) out of the 9 samples with high cell viability. The combination of cell viability and modulus provides two components of the criterion for optimal parameter selection.

**Figure 3:**
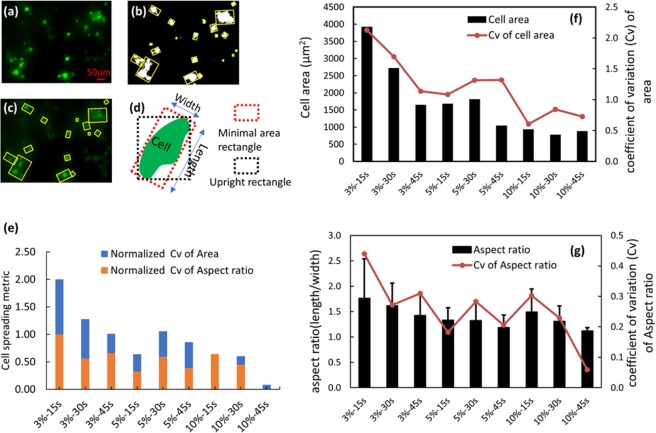
(**a**–**c**) Shows the cell boundary extraction procedure applied to live-dead cell assay images. (**d**) Schematic of boundary boxes with minimal area (dashed red box) and a upright rectangle (dashed black box) (**e**) Plot of the cell spreading degree, evaluated by the normalized Cv of cell area and normalized Cv of aspect ratio of minimal boundary box of each cell/cell aggregate. (**f**) Plot of measured cell/cell aggregate mean area and corresponding Cv in different samples. (**g**) Plot of aspect ratio of cell boundary boxes and corresponding Cv.

The third component is the degree of cell spreading, a critical indicator for cell culture environment evaluation. For certain types of cells, the more cell spreading observed the better the cellular environment^36–38^. To further identify the optimal printable GelMA concentration and UV crosslinking time, an image-based cell morphology analysis method was implemented to obtain a value for the degree of cell spreading. Figure 3(a–d) show the cell boundary extraction procedure, where the cells stained in green are segmented and encompassed by a rectangle with minimal area. The cells are measured within the GelMA constructs instead of near the surface (where all cells spread optimally).

A cell spreading degree metric has been developed by measuring the single cell area and cell boundary box aspect ratio from the fluorescent live cell images. The algorithm is described in the following steps: (1) The live cell image in RGB mode is transformed into HSV colorspace. (2) By properly defining a threshold in HSV colorspace, the cell image is converted to a grayscale image with cell area highlighted. (3) Further thresholding and morphological operations (image dilate and erode) are applied to convert the gray image to a binary image where the white pixels represent a cell or cell aggregates. (4) Contours of cells are determined from their binary images using the Python OpenCV library cv.findContours() method followed by the cv.contourArea() and cv.minAreaRect() to obtain the area and boundary box aspect ratio of each cell/cell aggregate. Figure 3(d) shows the boundary box of minimal area (white region in Fig. 3(b)) and the aspect ratio (defined as length/width) which are used in combination to evaluate the degree of cell spreading *D*_*spread*_. Figure 3(e) is the plot of *D*_*spread*_, which is evaluated by a sum of the coefficient of variation (Cv) of the average cell contour area and the aspect ratio of the boundary box with minimal area, for varying GelMA concentration/UV crosslinking time combinations. As the UV crosslinking time and GelMA concentration increase, the cells demonstrate decreased spreading (more rounded and smaller size). Figure 3(f,g) show plots of measured cell/cell aggregate area and aspect ratio of cell boundary boxes and their Cv in the 9 samples shown in Fig. 2(a). The standard deviation is not directly plotted along with the mean area which is much larger than the mean area value. The large standard deviation indicates that there are many different sizes of cells/cell aggregates. This is why the coefficient of variation (Cv) is advanced as a more suitable for the cell spreading metric. The degree of cell spreading decreases as GelMA concentration and UV crosslinking time increase, mirroring the same trend as cell viability.

The Cv is defined as the ratio of the standard deviation to the mean: ${C}_{v}=\frac{{\rm{\sigma }}}{{\rm{\mu }}}$, which shows the extent of variability in relation to the mean of the population. For example, the Cv of the aspect ratio decreases with increasing GelMA concentration, indicating that cells tend to be more spherical with increasing concentration. A larger value for the area Cv indicates spreading cells. The matrix that is used to define cell spreading degree is defined as:

2 $${D}_{spread}=C{v}_{{A}_{r}\_norm}+C{v}_{area\_norm}\,$$

The $C{v}_{{A}_{r}\_norm}$ is the normalized Cv of aspect ratio, and $C{v}_{area\_norm}$ is the normalized Cv of cell area. For example, the normalized aspect ratio and cell area are calculated as following:

3 $$C{v}_{{A}_{r}\_norm}=\frac{C{v}_{{A}_{r}}-\,\min (C{v}_{{A}_{r}})}{\max (C{v}_{{A}_{r}})-\,\min (C{v}_{{A}_{r}})}$$

4 $$C{v}_{area\_norm}=\frac{C{v}_{area}-\,\min (C{v}_{area})}{\max (C{v}_{area})-\,\min (C{v}_{area})}$$

From Table 1 we can easily identify the optimized parameters for GelMA printing as 3% GelMA and 45 s UV crosslinking time, where equation (1) has a maximum value of 0.830 representing the maximum value derived from the equation (1). Although the corresponding compressive modulus optimized from equation (1) is lower than that of the placenta-decidua^34^, this will enhance cell invasion in addition to enabling improved nutrient perfusion during long term culture.

**Table 1:** Optimization matrix for GelMA processing parameter selection.

GelMA parameters	3% (w/v)	5%(w/v)	10%(w/v)
15 s UV	No structure	0.493	0.434
30 s UV	No structure	0.547	0.415
45 s UV	***0***.***830***	0.478	0.294

The optimized parameters are applied in the cell invasion study sections. In summary, cell viability and cell spreading degree decrease as GelMA concentration and UV crosslinking time increase, thus the optimal parameters for the printing GelMA based bio-ink for the model are 3% GelMA and a 45 s UV crosslinking time.

### Multi-ring model for cell migration study

**Figure 4:**
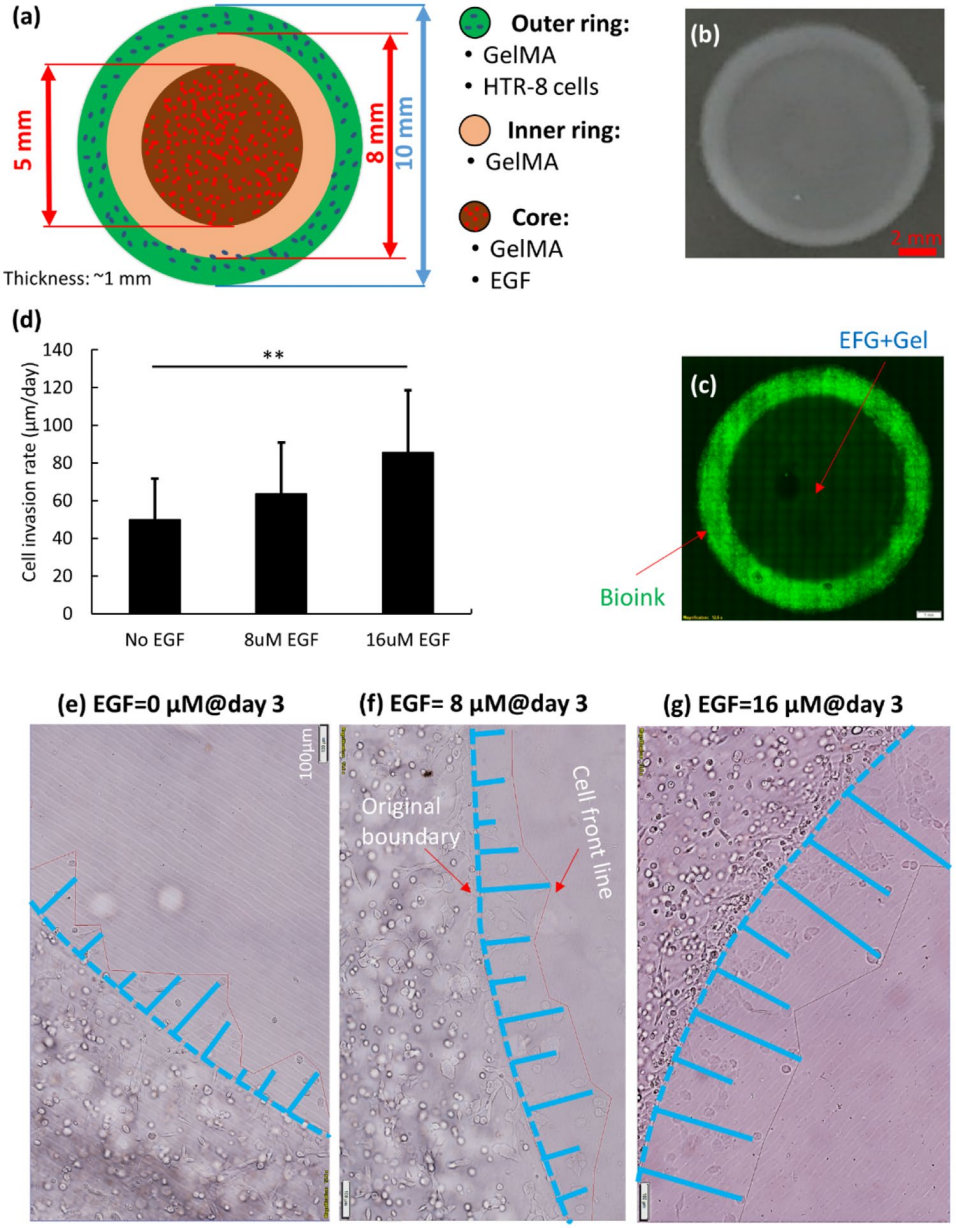
(**a**) Schematic of multi-ring model for the bioprinted cell-invasion study. (**b**,**c**) Show the grey-scale and color images of the printed ring model, the stained cells in the outer ring. (**d**) Measurement of the movement rate of the cell front in the first 3 days of culture. (**e**–**g**) Show sample regions measuring the rate of movement of the cell front.

In this section, we performed studies to identify the optimal model for characterizing trophoblast invasion. This model aims to show the concept of configuring a mesoscale geometry amenable to multicellular functional interrogation as part of a 3D cellular microenvironment that enables HTR-8/SVneo cells to invade through GelMA. The first model tested was the bioprinted multiple-ring model depicted in Fig. 4(a), where the HTR-8/SVneo cells were sequestered initially within a 1 mm thick (z axis) outer ring surrounding an inner ring (1.5 mm radial thickness) and an inner core with a diameter of 5 mm. The core contained EGF which, via diffusion over time, generated a chemoattractant gradient promoting cell invasion towards the core (2D multi-ring model). Figure 4(b) shows the color image of printed ring model while Fig. 4(c) shows the fluorescently stained cells in the outer ring. Importantly, when characterizing the cell front at different EGF concentrations over time, the cell morphology appears similar to that for cells cultured on a 2D surface, where the cells proliferate rapidly and spread. As shown in Fig. 4(c) the cell front moves at different rates under different EGF concentrations in first 3 days, but all are at a relatively high rate (≥50 µm/day). Even in the absence of EGF the cell front advances towards the center by day 3, demonstrating the measurement of rate of movement of the cell front.

### From 2D multi-ring model to 3D multi-ring model

**Figure 5:**
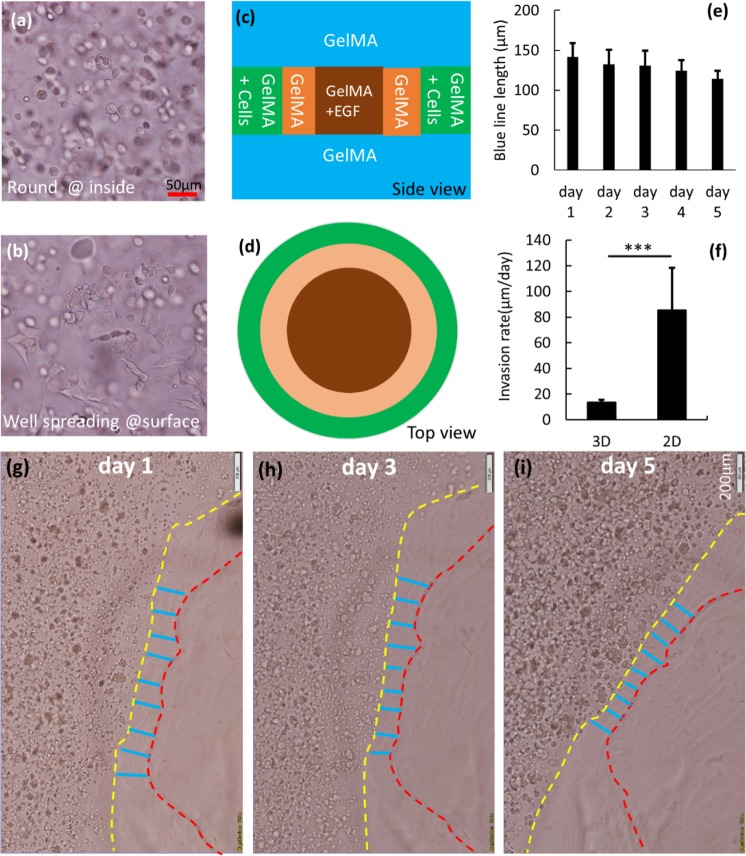
(**a**) Cell morphology within the GelMA construct. (**b**) Cell morphology near the surface of the GelMA construct. (**c**) Side view of encapsulated ring model design. (**d**) Top view of encapsulated ring model design. (**e**) Movement of cells within the GelMA construct. (**f**) Cell front movement rate in the 2D and 3D environments. (**g**–**i**) Sample region shows HTR-8 cell front line movement over 5 days culture.

In the previous section, a significant rate of movement of the cell front was observed near the surface in the 2D multi-ring model (Fig. 4(e–g)). However, close observation revealed that, compared to cells embedded within the GelMA construct, cells near the surface of the GelMA showed a greater degree of cell spreading (Fig. 5(a,b)). We hypothesized that cells near the surface of the GelMA demonstrate migration and proliferative behavior which promote significant movement of the cell front and subsume the invasive behavior. Thus, in this section, a modified ring model was developed with supplemental layers of cell-free GelMA on both top and bottom of the original ring model (3D multi-ring model). Figure 5(c,d) shows the schematic of the model, and Fig. 5(g,i) shows movement of the cell front over 5 days (16 µM EGF gradient). The red indicates the fixed (unmoving) reference line whereas the yellow line shows the cell front. The blue lines show the extent of cell invasion from the yellow line toward red line, over time, quantified in Fig. 5(e) for a 5d incubation. When compared to the cell migration in original ring model (2D multi-ring model), the movement rate in the 3D multi-ring model is much lower than the original (Fig. 5(f)). It is reasonable to infer therefore that the movement of the cell front in the 3D multi-ring model is attributable to cell invasion rather than migration and proliferation.

### From multi-ring model to multi-strip model

**Figure 6:**
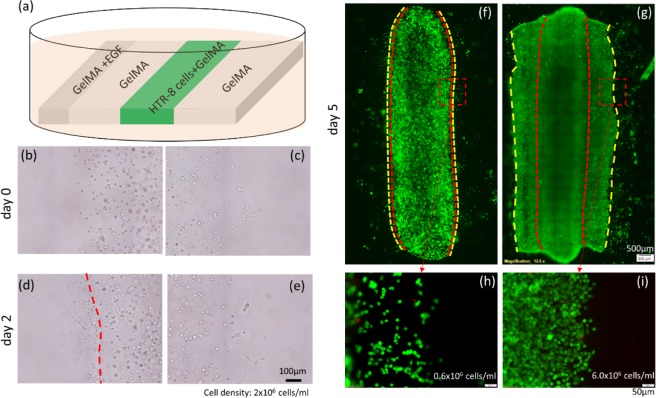
(**a**) Schematic of multi-strip model. (**b**,**c**) Cell/non-cell boundary at day 0. (**d**,**e**) Cell/non-cell boundary at day 2. (**f**) live cells at day 5 with printing density of 0.6 × 10^6^/ml. (**g**) live cells at day 5 with printing density of 6 × 10^6^/ml. (**h**) magnified cell boundary at printing density of 0.6 × 10^6^/ml (**i**) magnified cell boundary at printing density of 6 × 10^6^/ml.

Although cell front movement was studied in the two models (2D and 3D multi-ring model), there are some limitations to the ring model as a reliable platform for cell invasion studies. First, the ring model provides only one direction for cells to invade or migrate, lacking a comparable control group. In the 2D ring model, proliferation and the random movement of cells, may overwhelm the true invasive behavior of the cells. Second, in the 3D ring model, while the cell front movement rate (cell invasion) takes place at a low rate, cells are also subject to random migration, which cannot be measured. To this end, in this section, we developed a multi-strip model that enabled measurement of cell movement in two directions. First, 4 parallel strips (with dimension at ~2 mm width, and ~15 mm length) were printed and aligned side-by-side to create interfaces between the cell-containing strip and a cell-free strip (cell-gel interface) (2D multi-strip model; Fig. 6(a)). This enabled comparable evaluations of cell movement on both sides of the cell-containing strip at the same time. The EGF-GelMA, printed in the left-most strip, creates a chemoattractant gradient. Figure 6(b,c) show images of the cell-gel interfaces on day 0. Figure 6(b) corresponds to the interface closest to the EGF-GelMA strip and Fig. 6(c) is the interface on the other side of the cell-containing strip. Figure 6(d–e) show the cell-gel interfaces at day 2, where the cells aggregate on the EGF side, but maintain the same distribution as the non-EGF side. To investigate if this phenomenon is caused by cell proliferation, different cell densities were tested (0.6, 2.0, 6.0 × 10^6^ cells/mL). In Fig. 6(f–i), cell distribution at day 5 in two 2D strip models is shown. Figure 6(h,i) are magnified cell-gel boundary images of red dashed boxes in 6(f) and 6(g), with cell printing densities of 0.6 × 10^6^/ml and 6 × 10^6^/ml respectively. The cell front is represented as a yellow line and original position (reference) is labeled with a red line (cell front at day 0). These studies show that in the first 2 days, cell aggregation is observed on the EGF side when the cell density is 2 × 10^6^/ml or 6 × 10^6^/ml, but not in low cell density printing (0.6 × 10^6^/ml). Over time, the cells printed at low density (0.6 × 10^6^/ml) do not show strong migration outwards from the original strip whereas the cells printed at high density (6 × 10^6^/ml) show similar movement by the cell fronts on both EGF and non-EGF sides after 5d. The equivalent expansion of the cell front on both sides of the high-density strip suggests that these are proliferation effects rather than effects of the EGF gradient.

### Comparison of cell invasion in 2D and 3D multi-strip model

**Figure 7:**
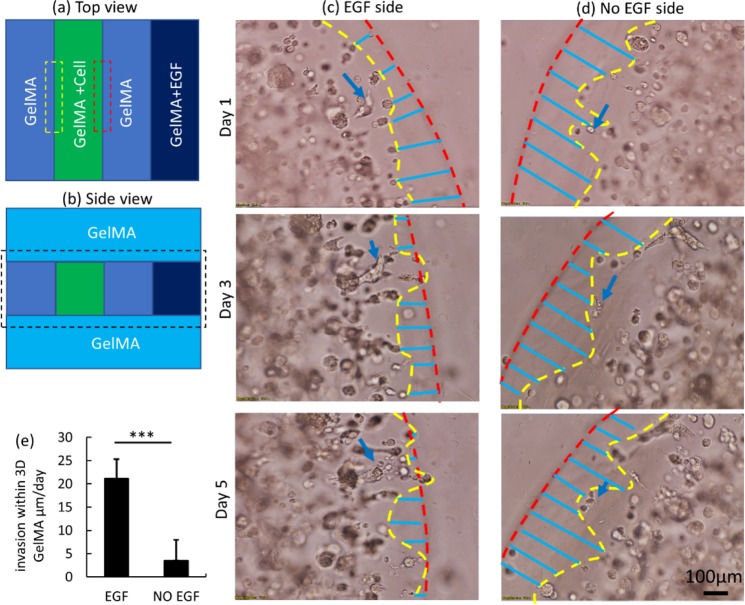
(**a**) Top view of multi-strip model design. (**b**) Side view of multi-strip model design. (**c**) Cell invasion in the 3D environment in the presence of EGF at days 1, 3 and 5 of culture. (**d**) Cell invasion in the 3D environment in the absence of EGF at days 1, 3 and 5 of culture. (**e**) Rate of invasion in the presence and absence of EGF.

It is important to note that the high rate of HTR8/SVneo cell front movement in the high- density, multi-strip model takes place at or near the surface of the cell-containing strip. In this section we therefore tested the effect of adding a GelMA layer on top and bottom of the multi-strip model (3D multi-strip model), ensuring that cells were in a 3D environment, where surface proliferation will not expand cell front outwards. Figure 7(a,b) show the schematic of the 3D multi-strip model covered with GelMA on top and bottom. Figure 7(c,d) show HTR8/SVneo invasion on days 1, 3 and 5 (cell density 3.5 × 10^6^/ml) in the presence (Fig. 7(c)) and absence (Fig. 7(d)) of the EGF gradient. The red line indicates the fixed reference and the yellow line shows the cell front. The blue lines indicate the distance between the yellow cell front and the red reference line. The extent of cell invasion is quantified in Fig. 7(e), where the effect of EGF on invasion is clearly apparent. The blue arrows indicate the same cell or cell aggregate on succeeding days. In the 3D multi-strip model, not only can the high rate of proliferative cell front movement be eliminated, but also certain cell/cell aggregates can be tracked over time, to ensure that measurement of cell front movement is a reliable indicator of HTR8/SVneo cell invasion.

## Discussion

One of the most challenging aspects of research into the placental function is the analysis of cell-cell interaction at the maternal-fetal interface. This paper describes a 3D bioprinted cell invasion model developed for the purpose of investigating the interaction processes. By combining the stiffness (compliance) of GelMA, cell viability, and cell spreading as metrics, we determined that the optimal GelMA concentration for the model was 3% and that the optimal UV crosslinking time was 45 s. Using the multi-ring model adopted from previous studies, we found that cells near the surface of the GelMA construct (2D multi-ring model) proliferate, spread and move faster than those embedded in a 3D environment (3D multi-ring model). Thus, movement of the cell front observed in the 2D model may be attributed, in large part, to cell migration rather than cell invasion. After covering the bottom and top of the ring model with a layer of cell-free GelMA (encapsulation), cell movement was significantly diminished. In the 3D multi-ring model however, cell movement is still a combination of the vectorial and proliferative types, and since the ring model has only one direction to measure cell movement, it is not possible to differentiate these elements. We therefore evaluated the multi-strip (2D and 3D) models, enabling simultaneous assessment of both unstimulated free movement and movement stimulated by EGF (invasion) and showed that the 3D multi-strip model could be used to measure both stimulated and unstimulated cell invasion. This well-characterized model paves the way for studies examining true trophoblast invasion in a multicellular model.

The optimal parameters determined for the hydrogel composition and formation are the best compromise between cell viability and structural requirements. Substitution of the LAP photoinitiator for the previously used Irgacure 2959^39,40^ enabled a significantly decreased crosslinking time (from 6 min to 45 s) with increased cell viability in a structurally less restrictive 3% GelMA construct. This is important, because the microstructure plays a vital role in cell invasion. We tried initially to use 5%w/v or 10%w/v GelMA for these studies, parameters adopted from the research of Kuo *et al*.^34^. However, we observed no cell movement within the hydrogel, only migration near the surface. Thus, it can be inferred that within the hydrogel, cellular movement is probably constrained by the dense GelMA microstructure, limiting their mobility. The elastic modulus is a good, indirect indicator of microstructure that shows a negative relationship between average GelMA pore size^41^ and elastic modulus, such that larger pore size equates to lower elastic modulus, enabling enhanced cell invasion. From the result-based point of view, we have optimized the process parameters for GelMA printing by utilizing the lowest elastic modulus that allows for retention of hydrogel shape. This has enabled cell invasion through the interior of the GelMA hydrogel. This does however raise an important question which we have not attempted to answer here. The elastic modulus measured for the placenta-decidua^34^ is substantially higher than the value optimized for use in our bioprinted constructs. This value, when used in bioprinting a GelMA construct, prevents cell movement in the interior of the hydrogel. It is not clear therefore, how the *in vivo* microstructure of the tissue with this high modulus value, which permits cell movement, differs from hydrogel with the same modulus value, which restricts movement. Further investigation is necessary to examine the relationship between elastic modulus, compliance and tissue microstructural characteristics, including extracellular matrix composition, cellular composition and microstructures such as capillaries.

A novel and very important modification to the model was the addition of the encapsulating, cell-free GelMA layers. Addition of these layers, while providing only a minimal barrier to equilibration with the culture medium, enclosed the cellular 3D environment, preventing the differential cell proliferation, spreading and movement which takes place at or near the hydrogel surface. Although these layers reduce the invasion rates, they ensure that the embedded cells are truly functioning in a 3D microenvironment and that we are measuring invasion rather than migration.

Another novel aspect of the studies performed here is the development of the multi-strip model. In this construct, the cells can move in two directions at the same time. This enables measurement of a control group of the same cells, separate from the EGF-stimulated group, compared to the unidirectional nature of the ring model. In the 2D multi-strip model, the factor which leads to the differential, unstimulated, cell movement rate is initial cell density. When cell density is low, cells near the GelMA surface are well spread out and are thus able to migrate and change their morphology freely during proliferation, leading them to occupy empty space without significant expansion. On the EGF side, cells aggregate (cell density increase) rather than causing movement of the cell front. As the initial cell density increases, cell front movement caused by free cell proliferation overwhelms cell invasion, and the newly generated cells have to expand on both sides. In the 3D multi-strip model, proliferation and unstimulated free cell movement are restricted by the cell-free GelMA layer, thus the cell movement measured on both the EGF and non-EGF sides shows significant differences. This shows the EGF gradient is clearly capable of stimulating cell invasion in the 3D environment.

While EGF diffusing from the GelMA-EGF strip might eventually reach the opposite side of the cell-containing strip, retarding free cell movement, this model is flexible. Thus, expansion of the cell-containing strip can be engineered such that EGF diffuses completely across the cell-containing strip only after cell invasion up the EGF gradient has been sufficient for measurement of invasion rate. Modification of the multi-strip model will accommodate alterations in cell density, chemoattractant type and gradient as well as the insertion of other strips containing structural or analytical components.

When 2D multi-strip model is covered with additional cell-free gel material encapsulant to produce the 3D multi-strip model, the cells can be tracked over time and invade at a rate around 21 ± 3 µm/day with an EGF chemoattractant gradient, where the cells on the control side invade only at a rate of 5 ± 4 µm/day. Although cell proliferation also occurs in the 3D environment, newly generated cells do not appear to advance the cell front, as evidenced by the tracking of individual cell/cell groups over time. These rates of invasion are similar to those we measured previously for modified BeWo and JEG3 choriocarcinoma cells^34^.

Our intention in these studies was not to mimic the properties of native decidual tissue. Rather, we wished to generate a functionally useable 3D matrix which demonstrates a closer resemblance to tissue than the 2D systems used previously. We have devised a method for integrating multiple variables, including measures of cell shape and spreading, to develop optimal 3D construct parameters and have used this method to generate the most advantageous conditions with respect to both mechanical and biological considerations.

In summary, this paper explores cell invasion *in vitro* using a novel bioprinted GelMA model. Compared to previous studies, this bioprinted model has various advantages including controllable geometry, mechanical properties, cell viability/morphology and cell density. The comparison of the 4 models tested here is shown in Table 2. We have optimized the concentration and UV crosslinking time. We have replaced the ring model with a much more flexible strip model which holds promise for testing multiple conditions in the same construct and eliminating inter-construct variability. We have added underlying and overlying cell-free gelMA strips which mitigate the problems of differential cell invasion rates at or near the surface of the hydrogel. These novel features provide significant functional advances which enable not only measurement of trophoblast invasion but the capability of examining cell-cell interaction during the invasion process.

**Table 2:** Summary of the 4 bioprinted models.

Comparison of 4 models	Multi-ring model	Multi-strip mode
2D	Pros: • Classical model, permits addition of other cell types in core ring. Cons: • No simultaneous control group • Differential proliferation and movement of cells near construct surface	Pros: • Capable to observe at two sides. Cons: • Differential proliferation and movement of cells near construct surface • EGF may diffuse across cell-containing strip.
3D (encapsulated with GelMA)	Pros: • Permits addition of other cell types in core ring. • Reduces unstimulated cell movement. Cons • No control group during observation.	Pros: • Reduces unstimulated cell movement and allows observation of cell movement from two sides. Cons: • EGF may diffuse across cell-containing strip.

## Methods

### Cell culture and viability assay

HTR-8/SVneo (HTR-8) cells (kindly provided by Dr. Charles Graham, Queens University, Kingston, Canada) were grown in RPMI-1640 medium containing 5% fetal bovine serum (FBS), 1% penicillin-streptomycin (P/S) solution, at 37 °C in a humidified 5% CO_2_ incubator. Before incorporation into the hydrogel constructs, cells were dissociated using 0.25% trypsin. Cells were then stained with Calcein-AM (2 μmol/L, Enzo), centrifuged and resuspended in the liquid hydrogel precursor. Cells were visualized after printing using a wide-field fluorescent microscope to assess viability.

### GelMA material preparation

GelMA macromer was synthesized according to a previously described method^42^. Gelatin (type A, 300 bloom, Sigma-Aldrich, St. Louis, MO, USA) was dissolved in phosphate buffered saline (PBS) at 50 °C at10% (w/v). Methacrylic anhydride (MA; Sigma-Aldrich, St. Louis, MO, USA) in solution form was added to the gelatin solution at a rate of 0.5 mL/min (50 °C) while mixing to reach a ratio of 0.6 g MA/g gelatin. The mixed solution was under stirring for 3 hr. (in the dark), the GelMA was formed and then it was diluted 5-fold with PBS (50 °C) and dialyzed against distilled, deionized water (DI water) at 40 °C for a week by using a 12–14 kD molecular weight cut-off dialysis membrane (Spectrum Laboratories Inc., Rancho Dominguez, CA, USA) to remove salts and excess free MA. Then GelMA foam was obtained after freeze-drying of the dialyzed solution for two days.

### Bio-ink formulation

To prepare the prepolymer solution, lyophilized GelMA was mixed with PBS (50 °C) for 10 min. Lithium phenyl-2,4,6-trimethylbenzoylphosphinate (LAP; Sigma-Aldrich, St. Louis, MO, USA) was mixed with the GelMA (final concentration 0.2% (w/v)) for 15 min at 50 °C, followed by addition of fibronectin (50 µg/mL). HTR-8/SVneo cells were mixed with the GelMA solution by gently pipetting, to avoid bubble formation. The bioink was then transferred to 4 °C for thermal gelation for 15 mins. Prior to printing, the bio-ink was warmed at a room temperature (21–22 °C) for 10 mins. If bubbles were observed upon loading into a reservoir syringe, the syringe was warmed to 37 °C to accelerate removal. For complete mixing, the bio-ink was maintained at room temperature and after printing was UV(UVP,UVL-18 365 nm UV Lamp, Analytik Jena US LLC), crosslinked according to experimental setting (15 s–150 s).

### 3D bioprinter system configuration

All cell-containing constructs were created using an in-house 3D bioprinter based on a customized 3-axis CNC machine mounted with an independently addressable printing head^7^. The toolpath was designed and transformed into G-code using Python, and then imported to the stand-alone open source software CNCjs to control the 3-axis translational platform. The bio-ink material formulation was loaded into a 3 mL syringe-based reservoir equipped with a 22 G nozzle (EFD, Inc.). The bio-ink deposition flowrate of 5 mL/hr. was controlled by modulating motor rotation speed. Stage travel speed was set at 8 mm/s^7^.

### Imaging and data analysis

An inverted bright field-fluorescence (IX83, Olympus) microscope was used to image the printed samples. Fluorescent images of printed cell-laden samples were processed using Cellsens software and Python (with OpenCV library) to yield quantitative measurements of cell viability through fluorescent image line-profile analysis. The statistical significance of measurements was determined using either Student’s t-test or one-way ANOVA analysis using GraphPad Prism 7 (GraphPad Software, La Jolla, CA). Differences were considered significant when p < 0.05 (*), p < 0.01 (**), p < 0.005 (***).

## Data Availability

All data generated or analyzed during this study are included in this published article.
